# Early Visual Processing is Affected by Clinical Subtype in Patients with Unilateral Spatial Neglect: A Magnetoencephalography Study

**DOI:** 10.3389/fnhum.2013.00432

**Published:** 2013-07-31

**Authors:** Katsuhiro Mizuno, Tetsuya Tsuji, Yves Rossetti, Laure Pisella, Hisao Ohde, Meigen Liu

**Affiliations:** ^1^Department of Rehabilitation Medicine, Keio University School of Medicine, Tokyo, Japan; ^2^ImpAct Team, INSERM U1028, CNRS UMR5292, Lyon Neuroscience Research Center, Bron, France; ^3^National Sanatorium Tama Zenshoen, Tokyo, Japan; ^4^Makuhari Ohde Eye Clinic, Chiba, Japan; ^5^Department of Ophthalmology, Keio University School of Medicine, Tokyo, Japan

**Keywords:** visual evoked magnetic field, pattern-reversal stimulation, attention network, diagnosis of unilateral spatial neglect, neglect subtypes, visual attention networks, viewer-centered neglect, stimulus-centered neglect

## Abstract

**Objective:** To determine whether visual evoked magnetic fields (VEFs) elicited by right and left hemifield stimulation differ in patients with unilateral spatial neglect (USN) that results from cerebrovascular accident.

**Methods:** Pattern-reversal stimulation of the right and left hemifield was performed in three patients with left USN. Magnetoencephalography (MEG) was recorded using a 160-channel system, and VEFs were quantified in the 400 ms after each stimulus. The presence or absence of VEF components at around 100 ms (P100m component) and 145 ms (N145m component) after stimulus onset was determined. The source of the VEF was determined using a single equivalent current dipole model for spherical volume conduction. All patients were evaluated using the behavioral inattention test (BIT).

**Results:** In response to right hemifield stimulation, the P100m and N145m components of the VEF were evident in all three patients. In response to left hemifield stimulation, both components were evident in Patient 3, whereas only the P100m component was evident in Patient 1 and only the N145m component was evident in Patient 2. Patient 1 exhibited impairments on the line bisection and cancelation tasks of the BIT, Patient 2 exhibited impairments on the copying, drawing and cancelation tasks of the BIT, and Patient 3 exhibited impairments on the cancelation task of the BIT.

**Conclusion:** These results demonstrate that early VEFs are disrupted in patients with USN and support the concept that deficits in visual processing differ according to the clinical subtype of USN and the lesion location. This study also demonstrates the feasibility of using MEG to explore subtypes of neglect.

## Introduction

Unilateral spatial neglect (USN) is a characteristic failure to explore the contralateral space of a brain lesion (Heilman et al., 1993). Although there have been many studies of the affected brain regions and pathological mechanisms of USN, general consensus is still lacking. This is largely because USN is a heterogeneous disorder with various subtypes that involve deficits in a variety of different spatial and representational cognitive processes, including personal or extrapersonal neglect and viewer-centered or stimulus-centered neglect, among others (Arene and Hillis, 2007). Most USN patients have a combination of the different subtypes. Therefore, it is difficult to find one common mechanism that underlies symptoms in all patients.

From a neuroanatomical perspective, brain lesions in a variety of regions have been emphasized as critical for USN, and there is controversy as to the one critical brain region. In particular, although several studies have suggested that lesions to the right inferior parietal lobe might be critical for USN (Vallar and Perani, 1986; Mesulam, 1999), another study found that lesions to the right superior temporal lobe were most common in USN patients (Karnath et al., 2001). Previous studies have also emphasized the role of fronto-parietal white matter disconnection in USN. Doricchi and Tomaiuolo (2003) found that damage to the fronto-parietal pathway caused chronic neglect, and Thiebaut de Schotten et al. (2005) found that inactivation of the right fronto-parietal connecting fibers during brain surgery caused stronger rightward deviation on the line bisection test. These findings suggest that fronto-parietal communication is essential for symmetrical visual processing, and indicate that spatial neglect is caused not by the dysfunction of a single cortical region, but by the disruption of large attention networks that include many discrete cortical regions.

In a recent study, Verdon et al. (2010) reported a relation between the clinical features of USN and the location of the brain lesion, highlighting the need to consider the different subtypes of USN when investigating the relation between lesion location and clinical characteristics of USN. However, prism adaptation and sensory stimulation ameliorate various symptoms of neglect (Luauté et al., 2006), suggesting that there may be a common mechanism underlying all subtypes of USN. Therefore, it is not clear if the clinical subtypes of USN share a common mechanism, or are mechanistically distinct.

It is largely accepted that USN is a high-order deficit and that sensory processing of contralesional stimuli remains intact (Heilman and Valenstein, 1979). Studies examining early (<200 ms) visual evoked potentials (VEPs) or event-related potentials reported that cortical activities are evoked by stimuli presented on the neglected side, thus supporting this view (Lhermitte et al., 1985; Vallar et al., 1991). However, recent studies have suggested that the early visual processing of contralesional stimuli is not normal in USN patients. The latency of steady-state VEPs was longer for contralesional than ipsilesional stimuli (Pitzalis et al., 1997), and early components of VEPs were delayed and of lower amplitude for left-side than for right-side stimuli in left USN patients (Di Russo et al., 2008). In addition, functional magnetic resonance imaging (fMRI) studies have reported that the right visual cortex of acute left USN patients was activated less by left hemifield stimulation than by right hemifield stimulation (Corbetta et al., 2005), and the response of the right primary visual cortex to left hemifield stimulation was reduced with high attentional load at fixation in left USN patients (Vuilleumier et al., 2008). These studies suggest that high-order attentional deficit can affect lower-order (early) sensory processing.

Visual processing of USN patients has been investigated using VEPs (Vallar et al., 1991; Di Russo et al., 2008), and fMRI (Corbetta et al., 2005; Vuilleumier et al., 2008). Although VEPs have higher temporal resolution than fMRI, they have a lower spatial resolution. Magnetoencephalography (MEG) is a non-invasive method of investigating human brain function that has been applied to the study of human visual processing (Cohen, 1968; Brenner et al., 1975). It can be used to measure changes in magnetic fields around the head that represent the electrical activities of neurons in the cortex, and has good potential for estimating source localization and temporal resolution of sensory processing. Therefore, using MEG to measure visual evoked magnetic fields (VEFs) can be a suitable method for elucidating the temporal and topographical process of early visual processing of USN patients. However, MEG has not yet been used to investigate visual processing in USN patients.

Visual pattern-reversal stimulation is a basic paradigm for the study of early visual processing (Halliday et al., 1972; Barnikol et al., 2006). Visual pattern-reversal stimuli evoke changes in the VEF (Nakamura et al., 1997; Hashimoto et al., 1999), and VEFs that are elicited by pattern-reversal stimulation have been well investigated (Nakamura et al., 1997; Hashimoto et al., 1999; Barnikol et al., 2006). In healthy subjects, VEFs have three components with latencies of 75–90, 100–120, and 145–160 ms, which are termed N75m, P100m, and N145m respectively (Nakamura et al., 1997; Hashimoto et al., 1999). Nakamura et al. (1997) reported that the N75m component was weaker than the P100m and N145m components. In addition, with dipole source analysis, reliable equivalent current dipoles (ECDs) of N75m elicited by hemifield stimulation were estimated in only 7 out of 12 sessions, even in healthy subjects (Nakamura et al., 1997). Therefore, N75m is not suitable for diagnostic evaluation of USN patients. Previous studies have suggested that the ECDs of P100m and N145m are located in or near the primary visual cortex (Nakamura et al., 1997; Hashimoto et al., 1999; Barnikol et al., 2006), but are in opposing directions, i.e., the ECD of P100m is directed medially and that of N145m is directed laterally (Nakamura et al., 1997; Hashimoto et al., 1999). Therefore, the direction and location of ECDs can be used to confirm the component under study.

It has been suggested that early visual processing in USN is affected by higher cortical dysfunction (Vallar et al., 1991; Corbetta et al., 2005; Di Russo et al., 2008; Vuilleumier et al., 2008). However, there are no studies that have compared visual processing across USN patients with different lesion locations or different neglect subtypes. The purpose of this study was to compare visual processing of USN patients between right and left hemifield stimulation, and to investigate whether lesion location or neglect subtype modulates visual processing.

## Materials and Methods

### Subjects

**Figure 1:**
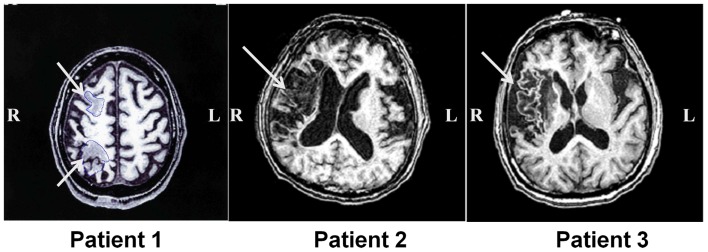
**Lesion location in patients with unilateral spatial neglect**. T1-weighted magnetic resonance images for all three patients with unilateral spatial neglect. White arrows indicate lesions.

Three patients with left USN were studied. This research was conducted in accordance with Declaration of Helsinki, and informed consent was obtained from each patient after the nature of the study was explained. Basic demographic characteristics of all patients are shown in Table 1. Magnetic resonance imaging (MRI) was performed with a GE Signa 1.5-T system (GE Yokogawa Medical Systems, Japan) and lesion locations were determined using T1-weighted images. The lesion of Patient 1 included the posterior parietal lobe and the posterior frontal lobe. The lesion of Patient 2 was in the posterior frontal lobe, temporal lobe, and extended to the temporo-parietal junction (TPJ). The lesion of Patient 3 was in the inferior frontal lobe and the temporal lobe (Figure 1).

**Table 1:** **Demographic characteristics of all patients**.

Subjects	Age	Sex	Disease	Time from onset (months)	BIT-C	BIT-B
Patient 1	54	Male	Rt. MCA infarction	4	108/146	64/81
Patient 2	70	Male	Rt. MCA infarction	4	82/146	45/81
Patient 3	57	Male	Rt. MCA infarction	1.5	99/146	68/81

*All subjects are right-handed*.

*BIT-C, conventional test of BIT; BIT-B, behavioral test of BIT; MCA, middle cerebral artery*.

### Evaluation of USN

The behavioral inattention test (BIT) is a battery that is commonly used to assess spatial neglect (Wilson et al., 1987). It consists of a six-item conventional test and a nine-item behavioral test. The cut-off values for spatial neglect are determined for each item and for the total score of the conventional and behavioral tests, and are determined as the average minus two standard deviations of the score of controls (Ishiai, 1999). Scores below the cut-off value indicate the presence of spatial neglect.

### Visual stimulation and MEG recording

Visual stimulation consisted of a reversal of a black-and-white checkerboard pattern. The luminance of the white squares was 200 cd/m^2^ and that of the black squares was 20 cd/m^2^, resulting in a contrast of 81.8%. The stimulus was back-projected onto a screen through a cylindrical duct (diameter 105 mm, length 600 mm) using a data projector (VPL FX-51, Sony, Tokyo, Japan) with a stable delay time (8.3 ms). The viewing distance was 15 cm. Patients lay comfortably in a supine position on a bed in a magnetically shielded room and watched the screen monocularly with the right eye. They were instructed to focus on a small red fixation point located in the center of the pattern. The background luminance of the shielded room was approximately 50 cd/m^2^. The pattern-reversal stimulation had 64 squares arranged in a matrix. The size of the stimulation was 20° × 20° and the inner edge was 1° lateral to the fixation point. The check size was 2.5° × 2.5°. The frequency of checkerboard reversal was 1 Hz. During each recording session, the stimulation was presented in the right or left hemifield, and an experimenter was sitting close to the patient to confirm eye focus. MEG was recorded using a whole-head 160-channel MEG system (MEGvison: Yokogawa Elec. Co., Japan). Five marker coils (Yokogawa Elec. Co., Japan) were placed on the skull for subsequent analysis of VEF source using MRI. MRI was performed within a week before or after MEG recording. T1-weighted images with 1.5-mm-thick contiguous slices were used for overlays, with the ECD sources determined from MEG data.

### Analysis

**Figure 2:**
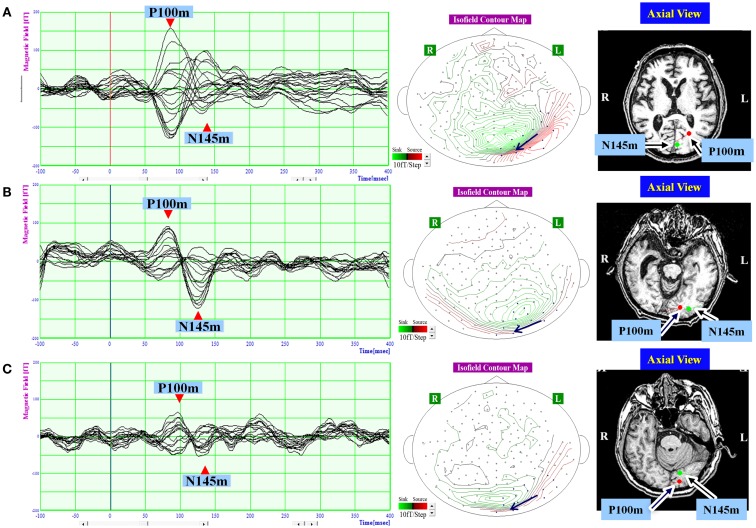
**The waveform and equivalent current dipole sources of visual evoked magnetic fields elicited by right hemifield stimulation in patients with unilateral spatial neglect**. Left: the waveforms of visual evoked magnetic fields (VEFs) in response to pattern-reversal stimulation of the right hemifield in Patient 1 **(A)**, Patient 2 **(B)**, and Patient 3 **(C)**. Waves detected by selected 16 magnetoencephalography recording channels are superimposed. Around 200 responses were averaged for each patient. Middle: the location of the 16 channels used to estimate ECD on the isofield contour map at peak time of P100m. In a contour map, green lines represent outward-going flux, and red lines represented inward-going flux. A black arrow indicates an expected location and direction of ECD. Small circles indicate distribution of recording sensors. Blue circles indicate selected 16 channels. Right: the equivalent current dipoles (ECDs) superimposed on axial magnetic resonance images. Red represents the P100m component of the VEF; Green represents the N145m component of the VEF. The dot represents dipole location, and the bar represents dipole direction. Both components were evident and were located in occipital lobe in all patients. The P100m component was directed medially, and the N145m component was directed laterally.

**Figure 3:**
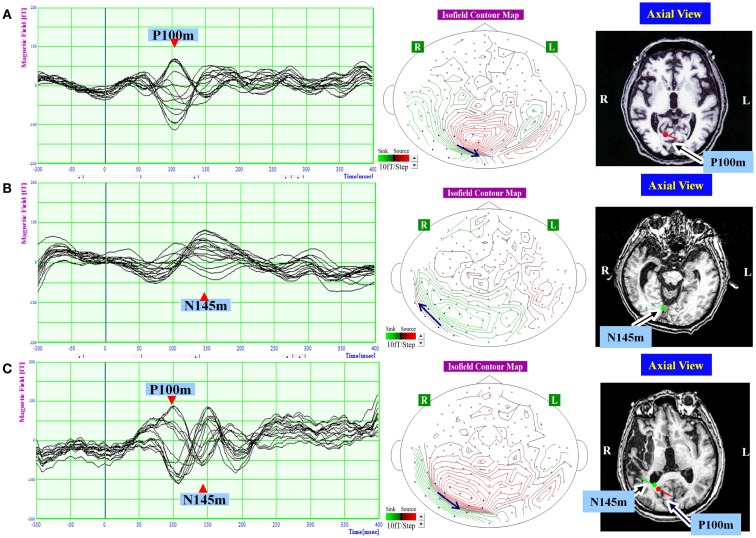
**The waveform and equivalent current dipole sources of and visual evoked magnetic fields elicited by left hemifield stimulation in patients with unilateral spatial neglect**. Left: the waveforms of visual evoked magnetic fields (VEFs) in response to pattern-reversal stimulation of the left hemifield in Patient 1 **(A)**, Patient 2 **(B)**, and Patient 3 **(C)**. Waves detected by selected 16 magnetoencephalography recording channels are superimposed. Around 200 responses were averaged for each patient. Middle: the location of the 16 channels used to estimate ECD on the isofield contour map at peak time of P100m **(A,C)** or N145m **(B)**. In a contour map, green lines represent outward-going flux, and red lines represented inward-going flux. A black arrow indicates an expected location and direction of ECD. Small circles indicate distribution of recording sensors. Blue circles indicate selected 16 channels. Middle: the equivalent current dipoles (ECDs) superimposed on axial magnetic resonance images. Red represents the P100m component of the VEF; green represents the N145m component of the VEF. The dot represents dipole location, and the bar represents dipole direction. The P100m component was evident in Patient 1 **(A)** and Patient 3 **(C)**. The N145m component was evident in Patient 2 **(B)** and Patient 3 **(C)**. All observed components were located in the occipital lobe. The P100m components were directed medially, and the N145m components were directed laterally.

Visual evoked magnetic fields were quantified using MEG data from 100 ms before to 400 ms after each stimulus. Around 200 responses were averaged for each patient. In healthy subjects, VEFs have three components: N75m, P100m, and N145m (Nakamura et al., 1997; Hashimoto et al., 1999). However, the N75m component is weak, and does not have reliable ECDs, even in healthy subjects (Nakamura et al., 1997). Thus, only the P100m and N145m components of the VEF were evaluated. The local responses from all 160 channels were superimposed, and we determined the times of the VEF peaks that occurred at around 100 and 145 ms visually. And then, the distribution of the magnetic field potential was represented in an isofield contour map according to the amplitude at each recording point at the determined time peak (Figures 2 and 3). In a contour map, green lines represent outward-going flux, and red lines represented inward-going flux. A source–sink pair indicated existence of a single-ECD source. Sixteen channels that covered the expected ECD location on the isofield contour map were selected at each time point for dipole source analysis. The root mean square (RMS) amplitude of the signal was calculated from the selected 16 channels, and a component was considered present if the peak RMS amplitude was above 40 ft.

To estimate location, intensity, and direction of each component, source analysis was based on a single-ECD model for spherical volume conduction. Using single-dipole theory, the ECDs were estimated at each time peak from the 16 channels that covered the occipital region and were localized on the MRI (Figures 2 and 3). Goodness-of-fit values greater than 90% were considered to indicate a good dipole model.

## Results

### VEF components

In response to right hemifield stimulation, P100m and N145m were evident in all three patients (Figure 2). The ECD of P100m was located in the primary visual cortex and directed medially, and the ECD of N145m was located near that of P100m and directed laterally (Figure 2). In response to left hemifield stimulation, P100m and N145m were evident in Patient 3, whereas only P100m was evident in Patient 1 and only N145m was evident in Patient 2 (Figure 3). In Patient 1 the ECD of the observed VEF was directed medially and in Patient 2 it was directed laterally, confirming that these were not the same components. The ECD of all detected components was located in the right occipital lobe around the primary visual cortex (Figure 3).

### USN symptoms

Behavioral inattention test scores are shown in Table 1. All patients obtained full marks on the line cancelation test, and all patients exhibited impairments (score below the cut-off value) on the letter and the star cancelation tests. Patient 1 also exhibited impairments on the line bisection test, and Patient 2 exhibited impairments on the copying and drawing tests (Table 2). The absent VEF component and abnormal components of the BIT are summarized for each patient in Table 3.

**Table 2:** **The profile of the conventional behavioral inattention test in all patients**.

Patient	Line cancelation	Letter cancelation	Star cancelation	Line bisection	Copying	Drawing
Patient 1	36	30*	34*	3*	3	2
Patient 2	36	8*	29*	7	1*	1*
Patient 3	36	14*	33*	9	4	3

*BIT, behavioral inattention test*.

*Scores under cut-off value.

**Table 3:** **The absent visual evoked magnetic field component, behavioral inattention test deficits, neglect components, and brain lesion location in all patients**.

Patient	VEF component	Deficit of BIT-C	Neglect component	Brain lesion
Patient 1	N145	Bisection cancelation	Perceptual/visuo-spatial	PPC, PFL
			Exploratory/oculo-motor	
Patient 2	P100	Copying, drawing cancelation	Allocentric/object-based	TPJ, TL, PFL
			Exploratory/oculo-motor	
Patient 3	not related	Cancelation only	Exploratory/oculo-motor	TL, IFL

*PPC, posterior parietal cortex; PFL, posterior frontal lobe; TPJ, temporo-parietal junction; TL, temporal lobe; IFL, inferior frontal lobe*.

## Discussion

Although many studies have investigated the cortical mechanisms of USN, the early visual processing of contralesional stimuli in USN patients is not well understood. To investigate early visual processing in USN, we compared the early components of VEFs elicited by right and left hemifield stimulation. Previous studies have suggested that the P100m and N145m components of the VEF are primarily generated in V1/V2 (Nakamura et al., 1997; Hashimoto et al., 1999; Barnikol et al., 2006). In this study, we determined criteria for evaluating the presence or absence of VEF components (RMS amplitude>40 ft and dipole source analysis goodness-of-fit>90%), and a response that did not satisfy these criteria was regarded as “absent.” Therefore, absence of a component does not necessarily mean “no response.” According to these criteria, the P100m and N145m components were both evident in response to pattern-reversal stimulation of the right hemifield in all three patients. However, the components of the VEF that were evident in response to left hemifield stimulation differed in the three patients. The three patients also had different neglect symptoms and different brain lesion locations (Table 3).

Early studies reported that early visual processing was intact in patients with USN. The early components of the VEP (<200 ms latency) were normal (Vallar et al., 1991), but the P300 component, which is related to attention, was abnormal for left-side information in left USN patients (Lhermitte et al., 1985). However, in the study of Vallar et al. (1991), USN patients were primarily diagnosed using cancelation and reading tests, rather than a using a standardized battery such as the BIT, and only two patients were evaluated by VEP. Therefore, they could not divide the patients into clinical subtypes. In addition, high-resolution recording system was not used in this study, there is no assurance that VEP components detected in response to left hemifield stimuli were really evoked in the right visual cortex. More recent studies performed using higher resolution recording systems suggest that early visual processing is affected in USN patients. Di Russo et al. (2008) found abnormalities in components of the VEP that occurred more than 130 ms after stimulus onset for stimuli located in the neglected side, whereas components of the VEP that occurred within 130 ms of stimulus onset were intact. Using fMRI, Corbetta et al. (2005) showed that the anatomically intact right striate cortex was less activated by visual stimulation than the intact left striate cortex in acute left USN patients. In USN patients with visual extinction, the P1 (80–120 ms latency) and N1 (140–180 ms latency) components of the event-related potential were absent or reduced for an extinguished stimulus with respect to a perceived stimulus located in the left visual field (Marzi et al., 2000; Driver et al., 2001). However, these studies did not consider the association between the subtype of neglect and the cortical activation observed in response to visual stimuli.

Attention and concentration increased the amplitude of VEPs elicited by pattern-reversal stimulation (Hoshiyama and Kakigi, 2001), and attentional load modulated the first (80 ms latency) and the second (108–120 ms latency) components of the event-related potential in early visual processing (Fu et al., 2009). These results suggest that higher cognitive function may affect early visual processing in the primary visual cortex. Furthermore, it has been suggested that the P100m and N145m components of the VEF are generated by independent and/or parallel activities of visual processing (Hashimoto et al., 1999; Barnikol et al., 2006), and the frequency and location of visual stimulation differentially affect early (equivalent to N75–P100) and late (equivalent to N145–P200) components of VEPs in healthy subjects (Parker and Salzen, 1977; Plant et al., 1983). These results suggest that attentional deficits may independently affect P100m and N145m. There is also evidence that higher cortical function may modulate early perceptual processing in USN patients. Valenza et al. (2004) reported that left primary somatosensory cortex responses to tactile stimuli on the “intact” right hand decreased when the hand was in the neglected left space, and Vuilleumier et al. (2008) reported that attentional load at fixation reduced right visual cortex responses to left hemifield stimuli in USN patients. These results suggest that early visual processing may be affected by higher cortical dysfunctions and by lesions in functionally related regions (Corbetta et al., 2005).

In this study, we found that the components of the VEF that were evident in response to left hemifield stimulation differed across the three USN patients. The three patients also had different symptoms, and different lesion locations. Although based on a small number of subjects, this is consistent with the recent suggestion that different subtypes of neglect are related to different cortical networks and/or regions (Hillis et al., 2005; Committeri et al., 2006). A recent neuroanatomical study supports this idea. Verdon et al. (2010) evaluated lesion location using voxel-based lesion-symptom mapping and revealed neural correlates for each component of neglect, namely the right inferior parietal lobule for the perceptive/visuo-spatial component related to the line bisection test, the right dorsolateral prefrontal cortex for the exploratory/visuo-motor component related to cancelation tests, and the deep temporal lobe region for the allocentric/object-centered component related to allocentric error in the Ota search test (Ota et al., 2001), which characterizes the object-based component of neglect. Although we did not use the Ota search test, the copying and drawing tasks of the BIT primarily evaluate the symmetry of figures that patients copy and draw, and may therefore be considered to represent the object-based component of neglect.

In a previous study, Di Russo et al. (2008) investigated early visual processing in USN patients, the majority of whom had lesions that included the parietal lobe. The results showed that visual processing 130 ms after stimulus onset was abnormal in the parietal lobe of USN patients, suggesting that low amplitude of N145m is related to parietal lesions. Combined with the finding of Verdon et al. (2010) that parietal lesions were associated with deviation in the line bisection test, these results suggest that a lack of N145m is related to parietal lesions and the perceptual component of neglect. Consistent with this proposal, we found that Patient 1 had a lesion of the posterior parietal lobe, no N145m VEF component in response to left hemifield stimulation, and exhibited strong deviation in the line bisection test.

In Patient 2, only one VEF component, at around 145 ms, was evident in response to left hemifield stimulation. This could be either a delayed P100m component or an N145m component. In previous studies, the ECD of P100m is always directed medially (Nakamura et al., 1997; Hashimoto et al., 1999); however, the ECD of the VEF component observed in Patient 2 was directed laterally. Therefore, we consider this to be N145m. Previous studies suggested that the frequency and location of visual stimulation differentially affected early (equivalent to N75–P100) and late (equivalent to N145–P200) components of VEPs in healthy subjects (Parker and Salzen, 1977; Plant et al., 1983). Therefore, P100m and N145m can be affected independently by higher cortical dysfunction. On the other hand, both P100m and N145m were present in Patient 3. This is compatible with previous reports that the early components of VEP were intact in USN patients (Lhermitte et al., 1985; Vallar et al., 1991). The lesions of Patient 2 and Patient 3 widely overlapped, making it difficult to discuss associations between lesion location and VEFs. However, only the lesion of Patient 2 extended to the TPJ; therefore, it is suggested that the absence of P100m is related to TPJ lesion and allocentric neglect. In addition, because all three patients exhibited impairments on the cancelation task, we suggest that the oculo-motor exploration necessary for the cancelation task was not related to the early VEF components.

Albeit from results of a single case, one possible hypothesis can be proposed to explain the VEF pattern of Patient 2. The check size of 2.5° × 2.5° in this study was larger than those of previous studies (Nakamura et al., 1997; Hashimoto et al., 1999). Previous studies demonstrated that amplitude of P100(m) increased in larger check size up to around 2° × 2° (Kurita-Tashima et al., 1991; Sahinoglu and Erar, 1999; Nakamura et al., 2000; Chen et al., 2005), while N145(m) decreased above 1° × 1° (Kurita-Tashima et al., 1991; Sahinoglu and Erar, 1999). It was also suggested that large checks activated peripheral vision more than central (foveal) vision (Nakamura et al., 2000) and large and small checks may preferentially activate different channels (Holder et al., 2010). Furthermore, the study that used large check size of 10.5° × 10.5° indicated activity in V5 complex area, as well as activity in V1/V2 area, contributed to P100m (Barnikol et al., 2006), while other studies that used smaller checks (<1°) showed that the ECD of P100m located in V1 area (Nakamura et al., 1997; Hashimoto et al., 1999). These findings suggest that dysfunction of TPJ may modulate activity of V5 area for peripheral vision that contributes to generation of P100m. However, N145m was preserved because it might be less sensitive to modulation of TPJ dysfunction than P100m. Further studies are needed to confirm this hypothesis.

A few limitations of this study warrant consideration. First, the number of subjects is small. Second, because there are no normal control subjects in this study, we cannot determine if the latencies and amplitudes of detected VEF components were intact. Third, MRIs were not recorded at the same day as MEG, and we did not use a standard brain image. In addition, because we use the single-ECD model for dipole source analysis, the effects of ECDs that may have existed at the same time as P100m and N145m were not considered, and we could not accurately estimate ECD location. Fourth, the check size of 2.5° × 2.5° is larger than that used in some previous studies (Nakamura et al., 1997; Hashimoto et al., 1999), although smaller than that used by Barnikol et al. (2006), and the signal strength of monocular stimulation may be smaller than that of binocular stimulation. These differences in stimulus condition may affect our results. However, because we stimulated both the right and the left hemifield with the same stimulus, we consider the differences between left and right hemifield stimulation to be reliable. Fifth, because of the MEG system’s technical limitations, devices such as electrooculogram could not be used to monitor eye movements and blinks, and we could therefore not remove the responses contaminated by eye movements and blinks.

Despite these limitations, we suggest that VEFs elicited by left hemifield stimulation are disrupted in USN patients. Our results support the concept that deficits in visual processing differ according to the clinical subtype of USN and the lesion location. USN is characterized by large heterogeneity in clinical aspects and neuroanatomical correlates (Arene and Hillis, 2007), and is considered to have multiple clinical components (Vuilleumier et al., 2008). Our study demonstrates the feasibility of exploring subtypes of neglect using VEFs measured by MEG, and this method can now be applied to larger groups.

## Conflict of Interest Statement

The authors declare that the research was conducted in the absence of any commercial or financial relationships that could be construed as a potential conflict of interest.
